# Women’s perceptions and reflections of male partners and couple dynamics in family planning adoption in selected urban slums in Nigeria: a qualitative exploration

**DOI:** 10.1186/1471-2458-14-869

**Published:** 2014-08-23

**Authors:** Joshua Oyeniyi Aransiola, Akanni Ibukun Akinyemi, Adesegun Olayiwola Fatusi

**Affiliations:** Department of Sociology and Anthropology, Faculty of Social Sciences, Obafemi Awolowo University, Ile-Ife, Nigeria; School of Public Health, University of the Western Cape, Cape Town, South Africa; Department of Demography and Social Statistics, Faculty of Social Sciences, Obafemi Awolowo University, Ile-Ife, Nigeria; Department of Community Health & Institute of Public Health, College of Health Sciences, Obafemi Awolowo University, Ile-Ife, Nigeria; Population and Reproductive Health Programme, Obafemi Awolowo University, Ile Ife, Nigeria

**Keywords:** Male Partners, Family Planning, Nigeria, Urban Slums

## Abstract

**Background:**

Nigeria is one of the countries where significant progress has not been recorded in contraceptive uptake despite decades of family planning programs while there are indications that slum dwellers may differ significantly from other urban dwellers in their sexual and reproductive behavior, including family planning uptake. This study therefore examined local notions regarding male partners’ involvement in family planning (FP) adoption by women in two selected urban slums areas in Nigeria – Ibadan (Southwest region) and Kaduna (Northwest region). Specifically, the study investigated women’s narratives about FP, perceived barriers from male partners regarding FP adoption by the women and how women negotiate male partners' cooperation for FP use.

**Methods:**

Sixteen FGD sessions were conducted with selected groups of men and women, stratified by sex, age group, and FP experience using a vignette to generate discussions. Sessions were facilitated by experienced social scientists and audio-taped, with note-taker also present. The transcribed data were analyzed with Atlas.ti software version 7. Inductive approach was employed to analyze the data. Reasons given for FP attitudes and use are presented in a network format while critical discourse analysis was also used in generating relevant tables.

**Results:**

The finding shows that women in the selected communities expressed desire for FP adoption. Three main reasons largely accounted for the desire to use FP: perceived need to space childbirth, family’s financial condition and the potential adverse effect of high fertility on the woman’s health. Male partners’ support for the use of FP by women was perceived to be low, which is due to misconceptions about FP and traditional pro-natalistic beliefs and tendencies. Mechanisms by which women negotiate their male-partner’s cooperation for FP adoption include seeking the support of the partner’s significant others and advice from older women.

**Conclusion:**

To significantly improve family planning adoption rates among urban slum dwellers in Nigeria, there is the need to specifically and specially target men alongside their female partners as well as other stakeholders who have significant influences at family and community level.

**Electronic supplementary material:**

The online version of this article (doi:10.1186/1471-2458-14-869) contains supplementary material, which is available to authorized users.

## Background

Globally, modern family planning methods have been recognized as means for addressing high births. In addition, condoms reduce the spread of HIV and other sexually transmitted infections (STIs). However, despite the wide availability of different modern family planning methods in most parts of the world, there are significant differences in the rates and patterns of adoption and utilization of the services [1–3]. These largely account for differences in the fertility rates, maternal mortality rates and prevalence rates of HIV and other STIs across different regions of the world [4–6]. Undoubtedly, family planning programs have played a major part in increasing contraceptive prevalence from less than 10% to 60% and reducing fertility from six to about three births per woman in developing countries over the past four-to-five decades[3]. Yet, contraceptive practice remains low and population growth high in half of the 75 larger low-income and lower-middle income countries, particularly those in sub-Saharan Africa [6].

Nigeria is one of the countries where significant progress has not been recorded in contraceptive uptake despite decades of family planning programs. The country’s fertility rate has consistently remained high over several decades, and is currently 5.7 children per women [7] as compared to 4.0 in neighbouring Ghana and 4.6 in Kenya, East Africa. Despite the high level of family planning awareness in Nigeria, current contraceptive use for modern contraceptives is just 10% [7]. There are, however, considerable variations across geographical areas and social strata. The contraceptive prevalence rate is significantly much lower in Northern Nigeria compared to the South. The rate of contraceptive use is lower among rural compared to urban dwellers, and among the poorer compared to richer population [7]. While demand for family planning remains low, unmet needs also remain high; the level of unmet needs for family planning is 20.2% among currently married and 15.8% among all women [7]. Besides, Nigeria also faces significant challenge in terms of HIV and AIDS; the country ranks as the third in the world in terms of the burden of people living with HIV [8].

Studies that have been carried out to assess factors associated with the low levels of family planning use in Nigeria have highlighted the role of religion, cultural barriers and various misconceptions about family planning, among others [9–11]. Inadequate support from male partners has also been identified in some studies as an important factor in family planning uptake by women [12]. Most studies, however, have focused on women and little attention given to men’s role in family planning. Most of the existing studies have, also, been purely quantitative in approach. Besides the limitations associated with such quantitative studies with regards to understanding the psychosocial interplay associated with family planning decision-making, very few of the existing studies [13, 14] have paid significant attention to the social stratification within the Nigerian urban population.

Spatial categorization suggests that an individual's geographical location, spatial entities and social boundaries are important determinants of behaviour. This view is well supported by the theory of environmental determinants of health behaviour [15, 16]. The underlining view of this line of thought is that individuals residing in *disadvantaged* locations are likely to exhibit some certain characters as influenced by the environment. Urban slum dwellers are typical examples of people within the social space of *disadvantaged* locations. According to UN-Habitat [17, 18], an urban slum is a heavily populated urban area with sub-standard housing and squalor. Focusing on urban slum has recently been recognized as an important agenda towards improving the health of the world’s population. Currently, about one billion people are estimated to be living in urban slums and the number is growing as rapid urbanization continues in low and middle income countries [19]. Nowhere is the problem of urban slum greater than in Africa, with about 62% of the region’s urban population living in slum conditions [20]. There are indications that slum dwellers may differ significantly from other urban dwellers in their sexual and reproductive behavior, including family planning uptake [21]. However, the contraceptive behavior of urban slum population in Nigeria has been poorly researched till date. This research aims to contribute towards addressing this important research gap [18, 22].

The role of male partners has recently become a growing concern with respect to family planning decision-making and practice [23, 24]. The influence of men in the areas of contraception, abortion, pregnancy and childbirth, as well as many other areas of maternal and child health has been clearly documented and remain relevant in most developing countries [25, 26], Also, the influence of patriarchal structure of the family and male dominance on family planning decision-making by women in urban cities, particularly in sub-Saharan Africa with its strong cultural rooting, has been severally reported [4, 27–30]. Little is however known about family planning-related attitudes and behaviour of urban slum men. Studies have documented women’s limited capacity to negotiate family planning use in Nigeria [12] and poor spousal communication on family planning [31, 32]. Yet, in the face of these challenges, there is little or no information on the various ways employed by women in slum areas to negotiate family planning use with their male partners. This is important for development of intervention strategies to promote active involvement of men in family planning adoption in slum areas. Particularly considering the high population growth rate projected for urban slums in the immediate future [33], it is important to address the research gaps relating to family planning dynamics in urban slums to inform relevant policy and program actions. The study examined local notions regarding male partner involvement in family planning in slum areas from the perspectives of women. It further investigated women’s narratives regarding family planning, and the various ways that women negotiate the cooperation of their male partners for family planning adoption. Overall, the study aims at providing information to inform the design of community level intervention to promote family planning adoption in Nigerian urban slums. Hence, the research questions is central around unravelling the dynamics involved in family planning decision making between partners in Nigerian urban slums.

## Methods

### Study setting and design

This study is qualitative in nature, with data collected through the use of focus group discussions (FGD) in two Nigerian cities -- Ibadan and Kaduna. Focus group discussion technique was adopted as the study aimed at exploring local notions and understanding the community norm in urban slums regarding contraceptive uptake and the dynamics of family planning negotiations within the community. Ibadan is the largest city in West Africa and is in South-west geo-political zone while Kaduna is the most cosmopolitan setting in Northern Nigeria and is in North-west geo-political zone of the country. The 2006 National Population and Housing Census [7] reported the population of Ibadan as 5,580,894 and that of Kaduna as 6,113,503. The indigenous and predominant population group in Ibadan is the *Yoruba* ethnic group while that of Kaduna is the *Hausa*. As a mark of their importance in Nigeria’s political development, the two cities served respectively as the seat of regional power for Western and Northern regions during the early post-independence period of three-region national structure. Ibadan currently serves as the capital of Oyo State and Kaduna as the capital of Kaduna State. The two cities remain arguably two of Nigeria’s fastest growing urban centres with the attendant challenge of increasing urban slum.

Within each of the two cities, an area regarded as urban slum was purposively selected based on predefined criteria relating to slums and the local knowledge of the areas. Agbowo community in Ibadan North local government area (LGA) was selected in Ibadan while Tudun Wada community in Kaduna South LGA was selected in Kaduna. The estimated population of Ibadan North is 308,119 while that of Kaduna South LGA is 402,731[34]. The two communities were selected as they represent typical slum areas and the population are predominantly of low socio-economic status. The study was carried out by the Centre for Communication Programs, Johns Hopkins Bloomberg School of Public Health, USA, in collaboration with the Population and Reproductive Health Programme (PHRP) Obafemi Awolowo University, Ile Ife, Nigeria, as part of the formative research for the Nigerian Urban Reproductive and Health Initiative (NURHI) project. NURHI project aims at understanding and addressing the constraints for family planning utilization in selected urban areas of Nigeria.

### Study participants

The participants were recruited at the community and health facility levels. Community entry was carried out through the traditional leadership structure of the communities, and the FGD participants were recruited through active interaction with the traditional leaders (*Baale* in Ibadan and *Mai-ungwa* in Kaduna) and the head of primary health care facility located in the communities. The community leaders helped in mobilizing the community members for active participation in the study. The participants were purposively selected to ensure that they met the study criteria: in this regard, a two stage procedure was used in selecting participants, starting with pre-FGD questionnaire administered to community members to identify those meeting the required criteria. The inclusion criteria were adult males (18–49 years) and adult females (18–35 years) who were regular resident in the community. Both married and unmarried adults as well as both users and non-users of family planning were included in the study population. Subsequently those meeting the study criteria and who consented to voluntary participation were included in the study.

Public and private health facilities offering family planning services which were within the urban slums selected were included in the larger study: this paper, however, focused only on women patronizing the facilities. The head of family planning units in each health facility helped in mobilizing clients for active participation in the study while the participants were selected based on the inclusion criteria mentioned above.

### Data collection

Eight FGD sessions were conducted in each of the selected urban slums, making a total of 16 FGD sessions. The groups were stratified by sex (males and females), age (younger women aged 18–24 years and older women aged 25 years and above), marital status (married and never married), and contraceptive status (users and non-users of family planning). The age group for adult married females was narrowed to 25–35 years in order to avoid both a married daughter and the mother belonging to the same FGD group thereby affecting the free participation of the younger generation in the group. This is because studies have indicated that the national average age at first marriage in Nigeria is 17 years and could be as low as 11 years in some states in the north [35–37]. It is important to note that the community leaders who could help identify married daughters and their mothers were not involved in the actual selection of the FGD participants in order to avoid their subjectivity intruding into the selection criteria. Never-married young males and females were included to provide an understanding of the attitudes of the next generation of adults to family planning adoption. Each focus group comprised of between 8 and 12 participants. The data was collected between April and December 2010. The groups are as presented in Table 1.

**Table 1:** **The focus groups involved in the study**

Males	Females
Never married males, 18–24 years (2 groups, 1 in Ibadan and 1 in Kaduna)	Never married females, 18–24 years (2 groups, 1 in Ibadan and 1 in Kaduna)
Young married males, 18–24 years (2 groups, 1 in Ibadan and 1 in Kaduna)	Young married females non-users of family planning,18-24 years (2 groups, 1 in Ibadan and 1 in Kaduna)
Older married males, 25–49 years (2 groups, 1 in Ibadan and 1 in Kaduna)	Young married females current users of family planning, 18–24 years (2 groups, 1 in Ibadan and 1 in Kaduna)
	Older married females non-users of family planning, 25–35 years (2 groups, 1 in Ibadan and 1 in Kaduna)
	Older married females current users of family planning, 25–35 years (2 groups, 1 in Ibadan and 1 in Kaduna)

The FGD guide developed for the study, based on the study objectives, was translated into the local languages (Yoruba and Hausa) of the participants through a two-way (back-translation) process involving language experts versed in each of the two languages. The focus group discussions involved the use of photo elicitation, vignette (story telling) and card ranking. This article reports on the vignette (story telling) part of the study. The vignette related the story of a 25 years old woman with two children (both girls). The first child was 4 years old while the younger was one and a half years old. The woman saw a poster at a local clinic about family planning methods that women can use to delay the birth of their next child. She thought it would be good to wait a while before her male partner and herself have their next child. She was, however, not sure how her male partner would feel about this and what to do. The story was related in the relevant cultural context and it led to some questions that examined attitude and challenges confronting urban slum women in taking vital reproductive health decisions, particularly with regards to family planning. Since the study was interested in community level intervention on attitudinal change to family planning, the questions were drafted to elicit community level constraints for family planning adoption. For instance, questions such as in this community, who usually starts the conversation about using family planning; and how involved do you think her husband will be in making the decision to use family planning and why, were asked. Participants were prompted on the questions. The group members were allowed to discuss and agree on issues raised. All FGD sessions were conducted by social scientists who are trained and experienced in qualitative research methods, including two of the authors (JOA and AIA). Data collection process continued until all the different important categories of slum dwellers relevant to the study were covered and saturation level appeared to be reached. Each session was audio-recorded, while an experienced note-taker was also present during each session.

### Data analysis

The analysis of the data was in two stages. A rapid analysis of the field notes was done first and this showed a clear pattern of the data and helped in developing themes and codes for the second phase of the analysis. In the second phase of the analysis, the audio-recorded data were transcribed verbatim in the local languages using standard transcription techniques, and the transcripts edited for accuracy. The edited data were then translated back to English by language experts. The transcripts were imported to Atlas.ti software (version 7) for qualitative analysis and themes were developed in line with the objectives of the study. Inductive approach was employed to analyze the data: in this process, the data were coded for new categories until the level of saturation was reached. The coding was carried out by a qualitative research expert who is a member of the research team. In order to guarantee intercoder reliability of the coding process, another qualitative research expert was consulted for additional coding until agreement and the level of saturation was reached by both experts.

The results are presented under different sub-themes as shown in the result section. The reasons given by the respondents with regards to family planning (FP) attitudes and use are presented in a network format while critical discourse analysis [38] presented in tables was also employed to support the network presentations. This is to help explain how certain things came to be said or done, and what has enabled and/or constrained what was spoken in the context of this study. This method does not aim at generalizability but rather shows how some phenomena are seen, emphasized and interpreted by the participants [39]. The numbers indicated in the cells (0–5) of Tables 1 and 2 are the number of quotations from each of the FGD sessions per coded themes. In addition to this, only interesting quotations summarizing the views of the participants were carefully selected to buttress the reports under the thematic issues discussed.

**Table 2:** **Women’s reasons for desiring family planning as indicated by male and female respondents**

Reasons women may desire family planning	Ibadan								Kaduna								TOTAL
	Males			Females					Males			Females					
	om	um	ym	Uf	Ofc	Yfc	yfn	ofn	Om	ym	Um	uf	yfn	ym	yfc	Ofn	
Child spacing	0	2	0	0	0	1	2	3	6	0	5	1	1	0	4	1	26
Children development	0	1	0	0	0	0	0	1	4	0	1	0	0	1	0	1	9
Family Economy	1	2	2	3	1	4	2	1	1	2	1	0	1	1	0	3	25
Family decision	0	1	0	0	0	2	0	0	0	0	0	1	0	0	0	1	5
Health problems	0	1	0	0	1	2	1	1	0	0	0	3	3	0	2	2	16
Imitate friends	0	1	1	0	0	0	0	0	0	2	0	0	0	1	0	0	5
Others reasons	0	0	0	0	0	0	0	0	0	0	1	1	0	0	0	0	2
Personal Career development	0	1	1	1	0	0	1	0	0	0	0	0	0	0	0	0	4
Satisfy male partner sexual desires	0	1	3	0	0	0	0	0	0	3	0	0	0	0	0	0	7
Want no more child	0	0	1	1	0	1	1	1	0	0	0	0	1	1	0	0	7
**TOTALS:**	**1**	**10**	**8**	**5**	**2**	**10**	**7**	**7**	**11**	**7**	**8**	**6**	**6**	**4**	**6**	**8**	**106**

**Keys:** om = old married males, ym = young married males, um = unmarried males, uf = unmarried females, ofc = older married females current users, yfc = young married females current users, ofn = old married females non-users, yfn = young married females non-users.

**Note**: The numbers in the cells (0–5) are the number of quotations from each of the FGD sessions per coded themes.

### Ethical considerations

The protocol for the study was approved, prior to the commencement of the study, by the Institutional Review Board of Johns Hopkins University and the Research and Ethics Committee of Obafemi Awolowo University Teaching Hospitals Complex, Ile-Ife, Nigeria, and the State Ministries of Health of Oyo and Kaduna States. Informed consent was obtained from all participants prior to the FGD: in this regard, an oral consent form was read to the participants, which covered the purpose of the study, the study procedures, possible risks/discomforts and benefits, as well as the issue of voluntary participation (Additional file 1). Among others, participants were explicitly informed that participation in the study was voluntary while participants were free to withdraw their involvements at any stage of the interview session. Appropriate steps were taken to guarantee the anonymity of the participants. For example, whereas community leaders were involved in mobilizing the community for active participation in the research, they were not involved in selecting the participants. Participants were selected by the researchers, who were complete strangers to the community. In addition, participants’ name or other personal identifiers were not recorded in the context of the research. Participants were given opportunity to ask any question and clarify issues before the FGD sessions commenced. Permission was obtained from the participants prior to audio-recording of the sessions. All informed consent documents, audio recordings and transcripts were kept under lock and key at the study research office while the manuscript had fully complied with RATS guidelines for reporting qualitative research.

## Results

### Family planning desire among urban slum women

**Figure 1:**
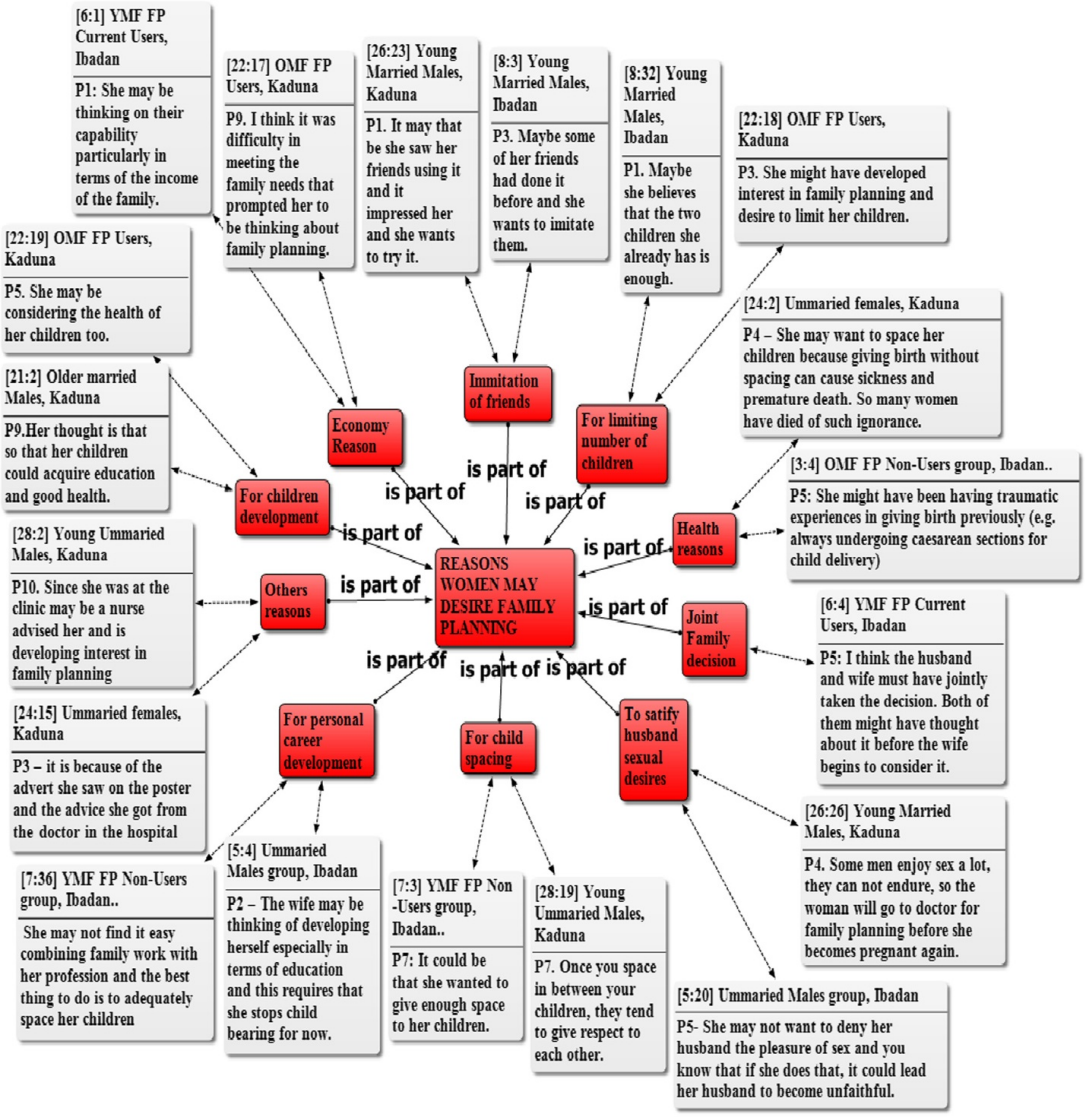
**Network of reasons women may desire family planning.** Key; OMF = Old Married Females, YMF = Young Married Females, FP = Family Planning. Note: The figures attached to the quotations refer to the number of the document and the paragraphs respectively, from where the quotations are cited in ATLAS.ti software. The one way arrows with “is part of” pointing to the main rectangular box at the middle shows that the reasons in the surrounding rectangular boxes are part of the broad reasons women may desire family planning. The two way arrows show that the quotations are selected examples of the reasons mentioned by the participants as it relates to the surrounding rectangular boxes.

Several reasons for desiring modern family planning methods by urban slum women were recorded during the study as shown in Figure 1. A major finding is that women desire to use family planning to limit the number of children and to be able to meet male partner’s sexual demand without the fear of pregnancy. Table 2 provides clear context within which family planning decisions are made in our focal slum areas. It presents more details as to women’s reasons for family planning desire from the perspectives of men and women. As Table 2 shows, women desire family planning for three main reasons: child spacing, family financial condition and their health conditions. On the other hand, a reason such as “family decision” was less mentioned by the participants.

The reasons underlying the women’s desire include the challenging economic situation of the family or economic aspirations, need to have time for personal development, desire to maximize child development, desire to maintain health and avoid potential negative health consequences of high fertility. In some cases, women’s desire to practice family planning was influenced by their desire to imitate friends who are living happily and progressing well in their careers as a result of limited number of children. Other reasons for desiring family planning include the couple’s decision on the desired family size, and the counseling received from health workers. The geographical variations in the pattern of family planning and the associated reasons are noteworthy. For example, whereas some women in Ibadan may want family planning on the basis of their desire for personal career development, this is less likely to be the case for women from Kaduna.

There were few occasions in which some women expressed the desire for large family (even when their male partners do not desire such). This is usually due to some personal belief regarding fertility or when they desire a child of a particular sex. In such cases some women could stop the use of family planning without the male partner’s consent to give an impression that the method failed. Others may back out of such relationship. Some participants narrated as follows:

*“There was a lady we both worked together. The couple had agreed to have two children. Eventually, the two children were boys. So the wife went for a third pregnancy which now turned out to be a girl. But the husband wasn’t happy with the wife. He even avoids carrying the new baby”.***Older married female, non-FP user, Ibadan.**

*“When I was still an apprentice, I met a man who wanted to marry me but said we will have only one child, I ran away from him because I will be sick if I don’t have many children – I mean as much as are in my womb. I don’t want to have any problem as a result of not giving birth to the number of children that I should have”*. **Young married female, current FP user, Ibadan.**

### Partners’ dynamics in fertility related decision-making

The dynamics of fertility related decision-making is presented in Table 3. A noticeable pattern in the initiation of discussion on family planning and decision-making regarding number of desired children is generally observed. Evidently, joint decision on family planning issues is less common than individual-led decisions. The participants reported that it is less common for the male partner to start family planning conversations, but they are usually the ones that take the final decision regarding the number of children.

**Table 3:** **Partners dynamics in fertility related decision-making as indicated by male and female respondents**

Who Usually start the conversation about family planning?	Ibadan								Kaduna								TOTAL
	Males			Females					Males			Females					
	om	um	ym	uf	Ofc	Yfc	yfn	ofn	Om	ym	Um	uf	yfn	ym	yfc	Ofn	
Male partner	0	0	1	0	0	2	1	0	0	3	0	0	1	0	0	0	8
Wife	0	2	1	2	2	1	1	2	0	2	0	0	1	2	0	5	21
Any of the partners	0	0	0	1	0	0	0	0	0	0	0	0	0	1	0	0	2
**Totals**	**0**	**2**	**2**	**3**	**2**	**3**	**2**	**2**	**0**	**5**	**0**	**0**	**2**	**3**	**0**	**5**	**31**
**Who usually decide the number of children for the family**																	
Both	0	0	0	0	1	1	1	2	0	0	0	0	0	0	0	0	5
Wife	1	0	0	0	0	2	1	3	0	0	0	1	2	0	0	0	10
Male partner	2	1	1	4	1	1	2	3	0	2	1	2	2	2	1	1	26
**Totals**	**3**	**1**	**1**	**4**	**2**	**4**	**4**	**8**	**0**	**2**	**1**	**3**	**4**	**2**	**1**	**1**	**41**

**Keys:** om = old married males, ym = young married males, um = unmarried males, uf = unmarried females, ofc = older married females current users, yfc = young married females current users, ofn = old married females non-users, yfn = young married females non-users.

**Note**: The numbers in the cells (0–5) are the number of quotations from each of the FGD sessions per coded themes.

Regional differences emerged between the two urban slum sites based on the comparison of the narratives obtained. The women in Ibadan, South-west Nigeria enjoy slightly better status than their counterparts from Kaduna in the northern part of the country. Women in Ibadan are more likely to be involved in joint decision with their male partners or be the decision-maker regarding the number of children the family should have compared to Kaduna women (Table 3).

The narratives reflected that the decision to stop the use of family planning does not solely rest with the woman, or the man. The male partner has the authority to instruct his wife to stop family planning even when the wife is not ready to have another baby. The woman can also stop the use of family planning even when the male partner support its use, for example when she desires to have another baby or she is experiencing side-effects with the use of contraceptives. The women indicated that male partners sometimes yield to pressure from the extended family (who feels the couple should have more children) and may thereby instruct his wife to stop family planning. For some men, the decision on family planning is linked to their perception regarding the availability of enough resources to cater for the number of children desired. In such cases, improvement in the male partner’s economic situation may make him desire to have more children, and thereby order his wife stop the use of contraceptives. Some participants affirmed as follows:

*“Some men are not strong willed. They can easily change their minds. Pressure from the extended family may force such a man to stop his wife (from continuing the family planning). Africa cherishes large family because of its link with prestige and honour in the society”.***Young married female, non-FP user, Ibadan**

*“Probably when the husband has gotten into sudden wealth and the economic status of the home has risen, the man may feel that they could afford to have more children now*” **Never married young female, Ibadan**

### Perceived husband-related dilemma regarding the use of contraceptives by urban slum women

**Figure 2:**
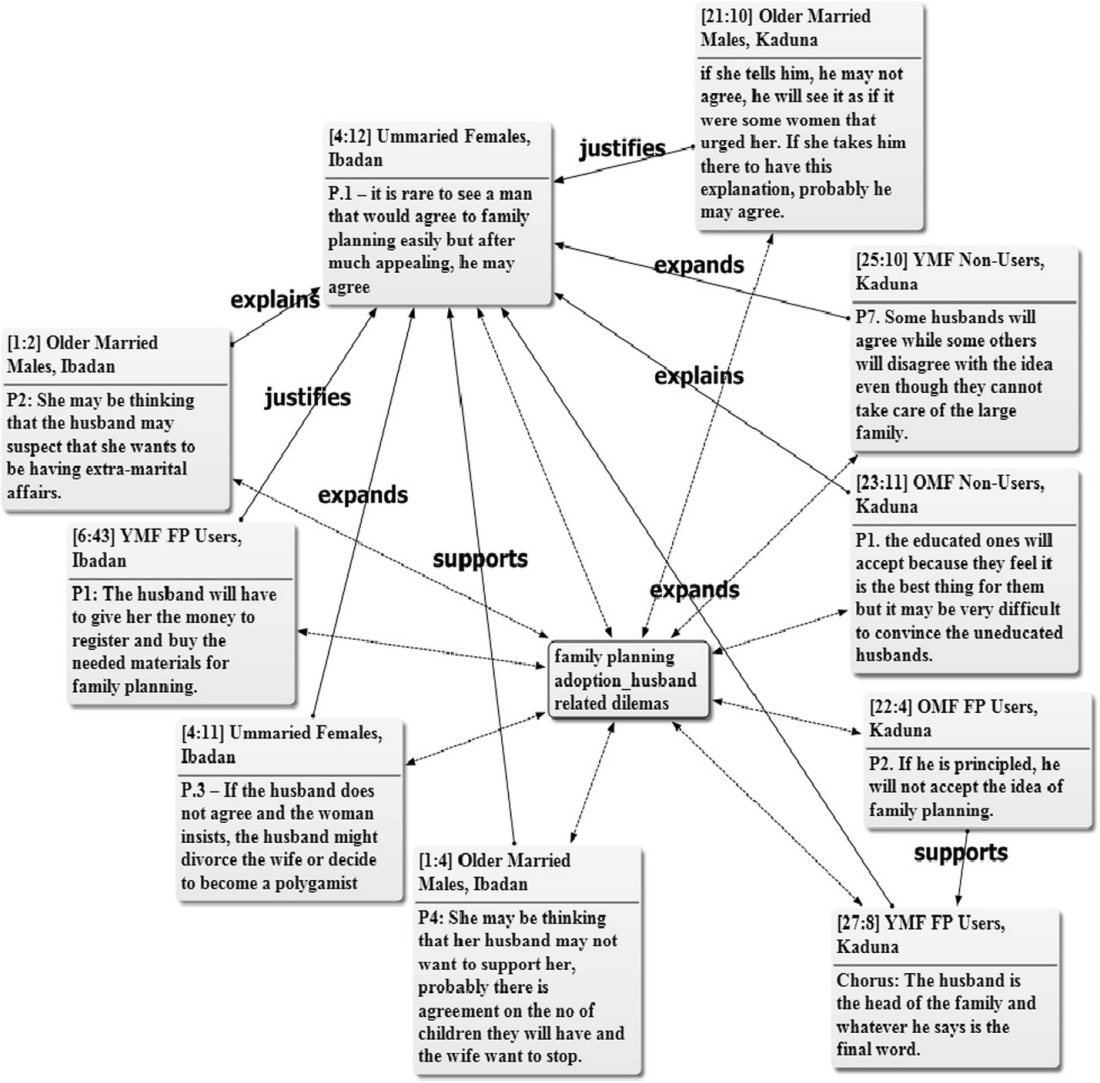
**Network of husband related dilemmas of family planning adoption.** Key: OMF = Old Married Females, YMF = Young Married Females, FP = Family Planning. Note: The figures attached to the quotations refer to the number of the document and the paragraphs respectively, from where the quotations are cited in ATLAS.ti software. The two way arrows show that the quotations are selected examples of the male partner related dilemmas mentioned by the participants across the FGD groups. Also, the quotation from unmarried females in Ibadan (i.e. 4:12) summarized the views of the participants across the focus discussion groups. Other quotations that help to explain, support, expand or justify this assertion are cited across other groups. Thus, helping to show how the thoughts of the different focus discussion groups are related.

The husband-related dilemmas for most women who desire family planning adoption were considerable. Majority of the participants expressed that although some men are interested in family planning, it is rare to see a man who may agree on family planning on his own until after much persuasion from the female partner. Even when the financial condition of the family cannot sustain large family, some male partners will not necessarily support their wives to use family planning because of the belief that it encourages infidelity or the fear that family planning may hinder the future plan of having more children. Most of the women in the urban slums also depend on their male partners for financial issues and may need financial support to obtain the family planning services (Figure 2). Across all the FGD sessions, the participants reiterated the importance of male partners’ approval before the wife will adopt family planning and indicated that the male partner’s decision is the final on the matter. Any attempt by the wife to do otherwise may results in maltreatment, divorce or the man may marry additional wife/wives. The common perspective of the participants in relation to the vignette were:

*“She (the woman) should go and seek her husband’s counsel and carry him along because the man should know and if there is any complication, the lady will not bear the brunt alone”* (**Chorus answer) Older married females, non-FP Users, Ibadan.**

*“She should discuss it with her husband and not any other person because if anything happens and the husband does not know, it can lead to misunderstanding in the home. The husband is the one taking care of her and she should not hide anything from him”*. **Never-married, younger female, Kaduna.**

The quotations below also revealed reasons given by participants as to why women should seek their husbands’ approval in adopting family planning:

***“****Because she is under her husband, if he says yes that is it, but if he did not accept she should not do it (family planning) to avoid trouble, or she will do it in secret”***Older married female, current FP user, Kaduna**

*“A woman after having three children went to the hospital without her husband’s consent to receive family planning methods. Her husband was expecting her to conceive again but she wasn’t. Later, the husband discovered while having sex with her one day that she had been using family planning method without his consent. He was angry. He even beat her. He told her to go and remove it and she later gave birth to one more child. If she had told her husband, she won’t have to go through that kind of experience.”***Older married female, non-FP user, Ibadan.**

### Perceived reasons as to why men may not support the use of family planning by their wives

**Figure 3:**
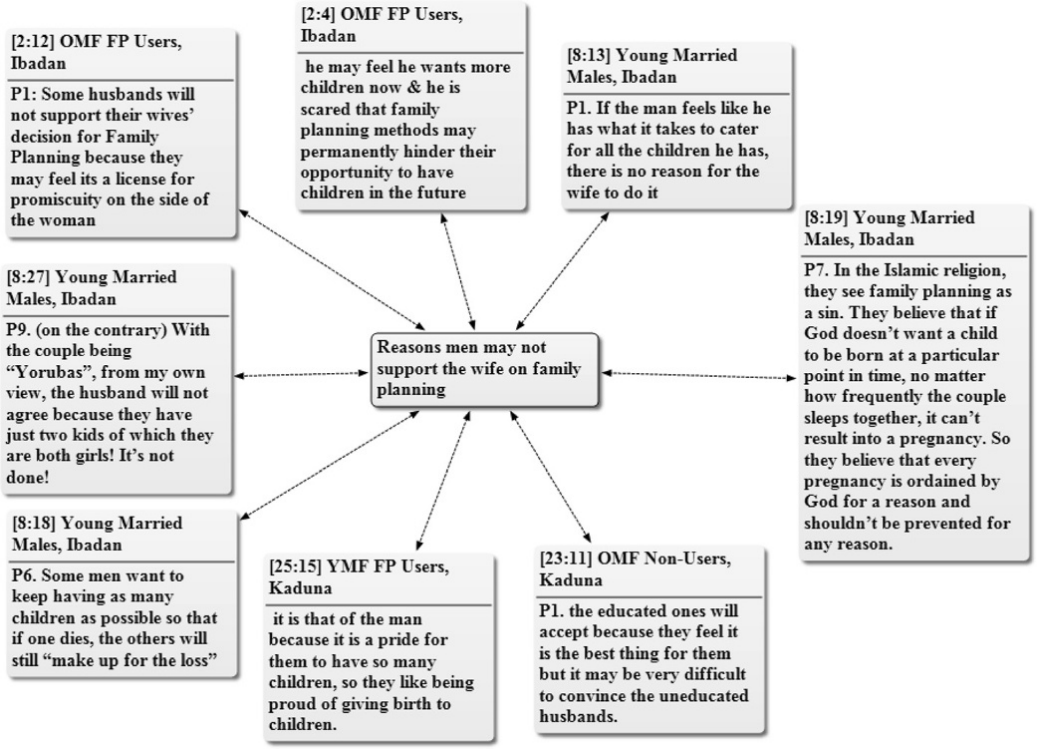
**Network of reasons men may not support their wives to adopt family planning.** Key: OMF = Old Married Females, YMF = Young Married Females, FP = Family Planning. Note: The figures attached to the quotations refer to the number of the document and the paragraphs respectively, from where the quotations are cited in ATLAS.ti software. The two way arrows show that the quotations are selected examples of the male partner related dilemmas mentioned by the participants across the Focus Discussion groups.

In addition to the reasons already mentioned, the FGD participants also indicated that formal education as well as socio-cultural beliefs may influence the male partners’ attitude towards the adoption of FP by their wives. Men with no formal education were mentioned as less likely to support their wives’ adoption of FP. On the other hand, men regard large family size with pride in traditional Nigerian societies. The patriarchal family structure, which lays emphasis on male child for the continuity of the family name, is also believed to be contributory to men’s attitude. As such, if all the children in the family are girls, the man may not support his wife to use family planning – with the desire to still have at least a son. Religious beliefs were cited as another factor that may play an important role in men’s attitude toward family planning. The high level of childhood mortality is another factor that figures in men’s attitude towards the practice of FP by their wives, as some men were said to believe that having large number of children will ensure that enough survive even if some die (Figure 3).

Although, the extended family members may have no direct authority in reproductive health decision-making, they are believed to be capable of influencing the husbands in many ways. The female participants noted that the extended family members, especially the mother-in-laws, have influence on their male partners either to increase or limit their number of children. The participants further reiterated that most of the extended family members favour high number of children but there are also few well- informed members who favour smaller family size. Some participants expressed their opinion thus:

*“If her (the wife’s) mother in-law once had problem with childbirth [delay] in her life, she won’t let her son’s wife do family planning because of her own ugly experience in the past*”. **Young married female, FP user, Ibadan.**

*“Other family members like the father in-laws, mother in-laws can influence the number of children a family has. For instance, where the family has all their children as girls, some extended family will convince the man to continue until he has male children because of the value placed on male children in our societies here. So many factors will influence the number of children a family has”.***Never married young female, Kaduna**

*“My mother-in-law told me to stop having children after my second child but I was not happy with the idea. However, now I know better. She had looked at the financial status of the family and knew that we can’t afford to have more children except we want them to come and suffer…so now I understand better”.***Older married female, non-FP user, Ibadan.**

### Wives’ perspectives on the roles expected of their husbands in family planning adoption

In the opinion of the female FGD participants, male partners should not only allow their wives the freedom to use FP, but should be actively involved in the process and provide support in a number of ways. The man, for example, should go with his wife to the health facility for counseling, provide the money for the services and give his wife the moral support necessary to use her contraceptive commodities faithfully. On the other hand, the women also identify that there are factors that may militate against the men playing the expected role. As a participant noted;

*“I am not sure most men will have the time to go with their wives to see any doctor. My own husband doesn’t have such time, he will ask me to go alone and see such doctor”.***Young married female, current FP user, Ibadan.**

### Ways in which women negotiate husbands’ cooperation for family planning adoption

Since men dominate reproductive health decision-making at the family level, it is important to understand the various ways by which women in urban slums negotiate their male partners’ cooperation to use family planning. This could give insights into possible strategies that could be used at community level to secure the support of men in the urban slums and other locations in family planning promotion.

**Figure 4:**
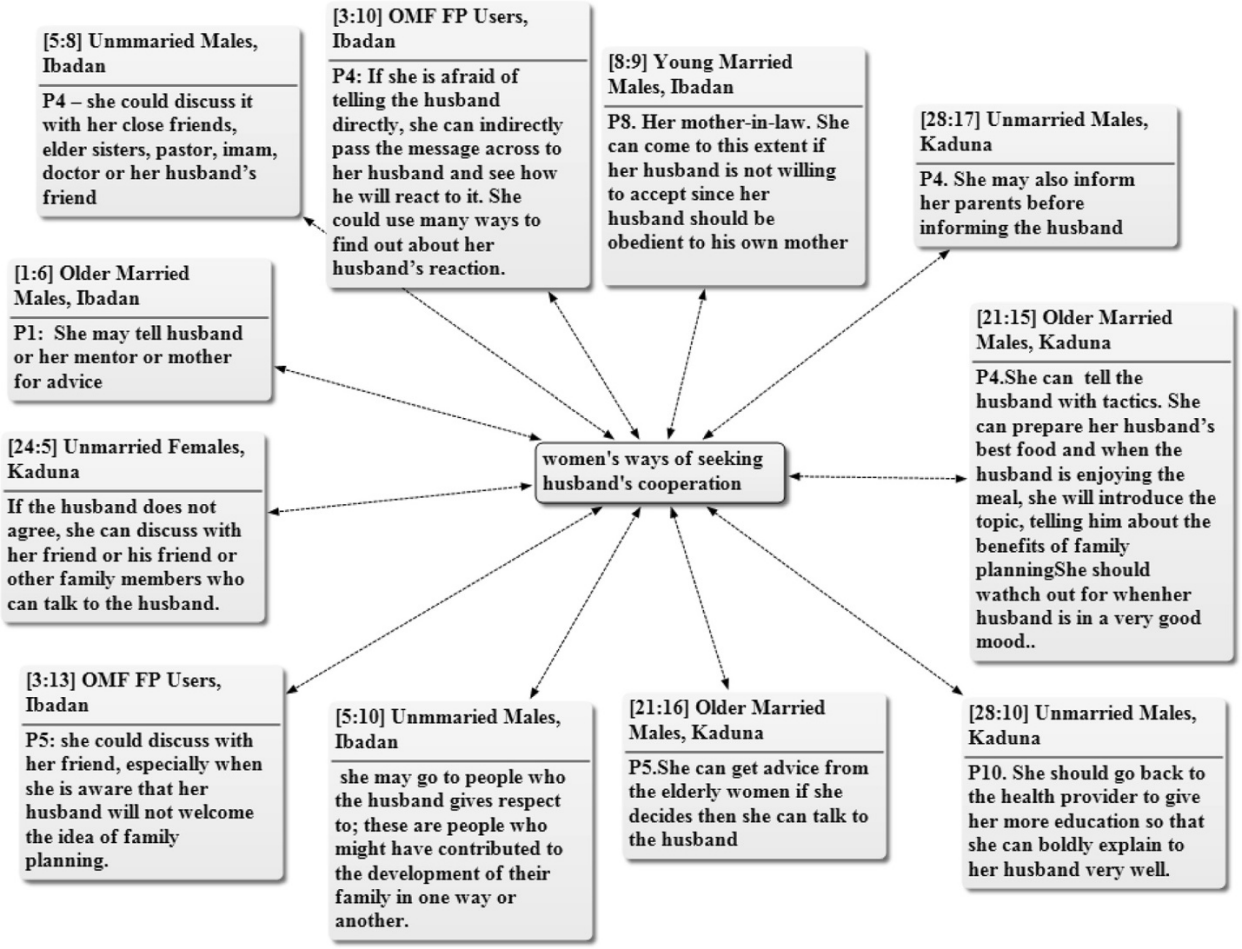
**Network of ways women negotiate their male partners’ cooperation for family planning adoption.** Key: OMF = Old Married Females, YMF = Young Married Females, FP = Family Planning. Note: The figures attached to the quotations refer to the number of the document and the paragraphs respectively, from where the quotations are cited in ATLAS.ti software. The two-way arrows show that the quotations are selected examples of different ways women use to seek their male partner’s cooperation as mentioned by the participants across the Focus Discussion groups.

The methods that women employ to get the support of their male partners for FP adoption include consulting with the man’s close friends who could influence him and mother-in-laws who favour family planning. The woman could also seek the advice of older women who she regards as mentors or other elderly women in the community to give her necessary advice. The assistance of religious leaders who are well predisposed to the use of family planning may also be sought in order to convince the male partners to allow their wives to use family planning. Figure 4 shows the various methods and individuals lobbied by the women to seek their male partners’ support towards the adoption of family planning methods.

## Discussion

This study examined the perception of women and local notions regarding couple dynamics in family planning adoption, including their reflection of the readiness of men to support their wives to adopt FP, and various ways that women negotiate the cooperation of male partners in two culturally diverse and prominent slum settings in Nigeria. Women expressed their desires for family planning on three main grounds: the need to space childbirth; family’s financial conditions; and, to protect the health of the wife. The women, however, believed that their male partners have poor attitudes to FP, based on a combination of culturally-influenced beliefs that favour high fertility and son-preference on the one hand, and misconceptions on the other hand. Our findings provide insights into how women tend to overcome the perceived barriers posed by various negotiation mechanisms – an area of inquiry that has received little research attention hitherto. Thus, while the women regard men as the final decision maker in issues of family planning and number of children that the family should have, they may employ some culturally-appropriate mechanism to influence men’s decision to enable the women practice FP. This finding challenges the assumption that men take critical fertility-related decisions and women simply obey; rather, the findings paint a picture of more iterative pathway and dynamic relationship in the FP decision-making processes of families. The findings also suggest that care must be taken in interpreting the results of quantitative studies that simply ask questions regarding who make the decisions regarding FP issues on the surface. Such questions and the answers received thereby need to be viewed through appropriate cultural lens to truly place them in context: an advantage of using qualitative approaches as we have done to better understand couple dynamics through exploration of women’s negotiation approaches.

The general perception of women in the urban slums that their male partners are not sufficiently supportive of contraceptive use is in line with some earlier findings that suggest that a high proportion of men have about FP [40, 41], although lower fertility rate in some patriarchal societies has been associated with men’s declining fertility desires [25]. Low male partner’s support potentially creates a lot of barriers for several other reasons. Women’s relative low economic power, which makes many of them financially dependent on their male partners, is a major challenge for women’s independent decision-making or even joint decision-making with their male partners. In this regard, women in the lower socio-economic class – which is the case with most women in urban slums – are even more vulnerable and may be less likely to have the freedom for independent reproductive health decision-making. The result of the 2008 NDHS also substantiates this, as the participation of women in their own health care is positively associated with their socio-economic level (as represented by the wealth quintiles) [7]. Thus, women’s participation in decision-making and their decision-making autonomy can be improved through financial empowerment. In general, there is the need for increased focus on women empowerment in the interest of improving contraceptive prevalence and reducing fertility in the urban slums of Nigeria. Financial empowerment for women in urban slum is particularly critical as they are generally poor and live under sub-optimal socio-economic and health conditions

Although women in the urban slums across different regions in Nigeria live under somewhat similar conditions, our findings suggest that there are differences between those in the South-west (Ibadan in this study) and those in the Northern part of the country (Kaduna in this study) in terms of their autonomy or participation in fertility- and contraceptive-related decision-making. Women in the urban slum area of Ibadan showed greater tendency towards autonomous or joint decision-making compared to their Kaduna counterparts. Similar pattern has been reported by the 2008 NDHS, which shows that while 68.9% of women in South-west reported participating in decision-making on their own health care, only 18.8% of women in the North-west reported such. The geographical disparity can largely be attributed to socio-demographic and cultural reasons. On the one hand, the cultural milieu of northern part of Nigeria is more restrictive of women’s participation in decision-making compared to those in the South. On the other hand, the educational level as well as the economic status of women in the North is generally lower than those in the South [42]. In addressing family planning issues in Nigeria, therefore, it is important to design programs in such a way that they are culturally relevant and acceptable to specific geo-political zones. Making contraceptives available free of charge or at highly subsidized rate, as recently decided by the Federal Government of Nigeria, also empowers poor women to easily cross the barrier facing them in the access and use of family planning.

The role of extended family members in shaping male partner’s decision to adopt family planning was severally identified by women in this study. Men of lower educational status or those in the younger age group may particularly be more susceptible to such influences. Studies in some other countries with a strong extended family structures carried out by Bawah et al. [43] and Kadir et al. [44] had similarly reported that extended family members especially the mothers-in-law (male partner’s mothers) have considerable influence on limiting family size. As such, it is important to include mothers-in-law and other aged members of the society in family planning communication interventions or other promotion and/or educational activities. Although, majority of these aged community members may not be sexually active, their influence on the younger generation may produce the desired reduction in fertility rate especially among the urban slum dwellers, if they are targeted for attitudinal change and as active promoters of family planning adoption. Such interventions may also help in combating the spread of sexually transmitted infections (STIs), including HIV, if condom is part of the contraceptive mix. For effectiveness however, such program needs to carefully identify some well-informed older community members, who could be targeted to serve as community resource persons to reach other members with contraceptive information and selected basic services.

Whereas literature has severally documented men’s low interest in family planning [40, 45] the question of how women negotiates their way through to FP adoption in resource-challenged environment, as indicated earlier, has not attracted significant research focus. The findings from our study provide some useful insight in this direction. It is interesting that a major approach used by women to get their male partner’s support for FP adoption is to send other men – who are their close friends – to their male partners. This finding provides a strong impetus for male-to-male outreaches and identifying male champions for family planning in various settings. Religious leaders with positive disposition towards family planning appears as potentially effective community-based FP champions – based on the women’s positive perception of their role in successfully brokering support for their FP intentions. It is interesting to note that women also identified FP-supportive older women – either in the forms of mother-in-laws or non-familial community-based older women – as possible allies in negotiating their male partners’ support for family planning adoption in strongly patriarchal societies. These findings suggest that contrary to what may be believed in many quarters, men in traditional settings are fairly susceptible to the influence of their significant others – males and females. Thus, while targeting men and involving them in FP programs evidently increase the likelihood of better outcomes in terms of family planning adoption practice [40, 45–47], there are other opportunities still in that men’s significant others matter in FP decision-making. These findings have significant programming implications: there is the need to simultaneously target and recruit both men and their significant others as active promoters and peer educators in the context of family planning intervention programs in urban slum settings, and probably in other settings as well. Such programming approach has the potentials to accelerate positive attitudinal change towards family planning and FP adoption among men in urban slum, thereby breaking a major barrier that women perceive to stand in their way vis-à-vis FP adoption.

It is important to note that the results of this study does not provide information about family planning adoption at individual level and cannot be generalized since it relied on qualitative research technique, and specifically, FGD. It, however, provided information on male-related perceived constraints inhibiting family planning adoption at community level, from the perspectives of women. Overall, the results of this study have provided important information on family planning adoption in Nigerian urban slums, which has been poorly researched till date, thereby contributing towards addressing the gap in reproductive health studies. Specifically, the study has explored common local notions among women with regards to their own desire for family planning, the balance in decision-making between them and their male partners, perceived men’s negative attitude to FP, and mechanisms for successfully negotiating FP adoption with their male partners. In addition, this study provides insight regarding women’s expectation of their husband in family planning matters, and the challenges that may stand in the way of men in meeting such expectations.

## Conclusions

The findings of this study indicate that women in slum areas in Nigeria hold the notion of poor attitude on the part of men regarding family planning which, in turn, impacts negatively on women’s ability to adopt and use family planning. The degree of women’s participation in family planning decision-making within Nigeria’s broad traditional patriarchal setting are mediated by a number of factors, including local culture and community norms, socio-economic status, family setting and influence of extended family members, as well as individual characteristics. In general, Nigerian women in urban slum settings hold the belief that women must not take family planning decisions on their own, but rather consult with their male partners irrespective of men’s perceived attitude. However, men’s negative attitude is not regarded by the women with a view of finality as they may explore a number of avenues involving different categories of people to broker negotiations and win their male partners’ support for FP adoption. Overall, our findings provided empirical information and evidences that can be used in improving FP programming in Nigerian’s urban slums. The findings strongly suggest that for the greatest impact, family planning programs must strategically target men alongside women of reproductive age, and should also simultaneously target older community members and other stakeholders who can have influences at family and community levels and serve as local champions for FP promotion. Yet, it is important to recognize that the degree to which different factors influence FP behaviours can vary significantly in diverse settings, despite some similarities in locally held notions. Thus, in designing and implementing family planning programs, care must be taken to ensure that they are culturally relevant and informed by the evidences specific to each local environment.

## Authors’ information

JOA is a Senior Lecturer in the Department of Sociology and Anthropology, Obafemi Awolowo University, Ile-Ife, Nigeria. He is currently a visiting researcher in HIV and AIDS Research Centre, School of Public Health, University of the Western Cape, Cape Town, South Africa. He has a PhD degree in Sociology and Anthropology. His main areas of research interest are women and children’s rights, including reproductive health rights.

AIA is a Senior Lecturer in the Department of Demography and Social Statistics, Obafemi Awolowo University, Ile-Ife, Nigeria. He has a PhD degree in Demography and Social Statistics. His main areas of research interest are reproductive health, ageing and migration.

AOF is a Public Health Physician and Professor of Public Health and Community Medicine at the Obafemi Awolowo University, Ile-Ife, Nigeria. He is currently the Director of the university’s Institute of Public Health as well as the Population and Reproductive Health Programme. His main areas of research interest are adolescent health and development, and reproductive health.

## Electronic supplementary material

Additional file 1: **ORAL CONSENT SCRIPT: Focus Group Discussions.** (DOCX 22 KB)
