# Supplementary material for: Women’s perceptions and reflections of male partners and couple dynamics in family planning adoption in selected urban slums in Nigeria: a qualitative exploration
Source: BMC Public Health. 2014 Aug 23;14:869. doi: 10.1186/1471-2458-14-869 (PMC4165936; doi:10.1186/1471-2458-14-869)
Supplement: Supplementary file 1 — Additional file 1: ORAL CONSENT SCRIPT: Focus Group Discussions. (DOCX 22 KB) [file 12889_2012_7034_MOESM1_ESM.docx]

#### **Additional file 1**

#### **ORAL CONSENT SCRIPT**

**Focus Group Discussions**

**PURPOSE**

Good Morning/afternoon/evening. Thank you for taking the time to come to this meeting today. My name is                                                           _____ . I represent the [name of local non-governmental organization conducting the study]. We are working with the Johns Hopkins University to conduct group discussions family aspirations and the use of family planning in Nigeria.

**PROCEDURES**

I will ask you and others in the group questions about general opinions and perceptions of families and family planning in the community. It is important that you participate from the beginning to the end and that you be honest in your answers. However, you should feel free to leave the group at any point during the discussion.

The talk will take approximately 2 hours and refreshments will be provided at the end.

**RISKS/DISCOMFORTS**

Only the participants in the group and team members that are conducting this study will have access to to the information that you share with us today. People that participate in the discussion today may share with others outside what we speak here today, but you can be sure that the members of the study team will not share with others what we discuss here today. It is worth telling you that this has not been reported following studies such as this. Only members in the study will have access to this information. However we cannot guarantee that some of you could speak to others about our conversation today. For the study reports, no names of participants will be used. So that I do not forget important information about what we talk about today, I am going to audio record the discussion. If you prefer, you can choose another name or symbol, which we will use to call you during the meeting.

**BENEFITS**

Although there is no direct benefit to you from being in this study, the information we gather from you and other community members will help us develop and improve the programs we will support in your community.

**VOLUNTARY PARTICIPATION**

You do not have to agree to be in this study, and you may change your mind at any time.

If you have any other questions about this study after we finish, you can contact (provide contact card; Name, title and phone number)

Do you have any question that you would like to ask me now about the study?

Do you agree to participate in this group discussion?

Do you agree to have the group discussion audio recorded?

“I have read the consent form completely before the participants and they voluntarily agreed to participate in the study.”

Signature of Fieldworker

Date
